# Ex vivo expanded SSEA-4+ human limbal stromal cells are multipotent and do not express other embryonic stem cell markers

**Published:** 2012-05-14

**Authors:** Moon Nian Lim, Noor Hamidah Hussin, Ainoon Othman, Thiageswari Umapathy, Puteri Baharuddin, Rahman Jamal, Zubaidah Zakaria

**Affiliations:** 1 Stem Cell Laboratory, Haematology Unit, Cancer Research Centre, Institute for Medical Research, Kuala Lumpur, Malaysia; 2 Department of Pathology, Faculty of Medicine, Universiti Kebangsaan Malaysia, Kuala Lumpur, Malaysia; 3 Department of Ophthalmology, Hospital Kuala Lumpur, Malaysia; 4 UKM Medical Molecular Biology Institute (UMBI), Kuala Lumpur, Malaysia

## Abstract

**Purpose:**

The presence of multipotent human limbal stromal cells resembling mesenchymal stromal cells (MSC) provides new insights to the characteristic of these cells and its therapeutic potential. However, little is known about the expression of stage-specific embryonic antigen 4 (SSEA-4) and the embryonic stem cell (ESC)-like properties of these cells. We studied the expression of SSEA-4 surface protein and the various ESC and MSC markers in the ex vivo cultured limbal stromal cells. The phenotypes and multipotent differentiation potential of these cells were also evaluated.

**Methods:**

Limbal stromal cells were derived from corneoscleral rims. The SSEA-4^+^ and SSEA-4^-^ limbal stromal cells were sorted by fluorescence-activated cells sorting (FACS). Isolated cells were expanded and reanalyzed for their expression of SSEA-4. Expression of MSC and ESC markers on these cells were also analyzed by FACS. In addition, expression of limbal epithelial and corneal stromal proteins such as ATP-binding cassette sub-family G member 2 (ABCG2), tumour protein p63 (p63), paired box 6 (Pax6), cytokeratin 3 (AE5), cytokeratin 10, and keratocan sulfate were evaluated either by immunofluorecence staining or reverse transcription polymerase chain reaction. Appropriate induction medium was used to differentiate these cells into adipocytes, osteocytes, and chondrocytes.

**Results:**

Expanded limbal stromal cells expressed the majority of mesenchymal markers. These cells were negative for ABCG2, p63, Pax6, AE-5, and keratocan sulfate. After passaged, a subpopulation of these cells showed low expression of SSEA-4 but were negative for other important ESC surface markers such as Tra-1–60, Tra-1–81, and transcription factors like octamer-binding transcription factor 4 (Oct4), SRY(sex determining region Y)-box 2 (Sox2), and Nanog. Early passaged cells when induced were able to differentiate into adipocytes, osteocytes and chondrocytes.

**Conclusions:**

The expanded limbal stromal cells showed features of multipotent MSC. Our study confirmed the expression of SSEA-4 by a subpopulation of cultured limbal stromal cells. However, despite the expression of SSEA-4, these cells did not express any other markers of ESC. Therefore, we conclude that the cells did not show properties of ESC.

## Introduction

The cornea is the major refractive element of the adult eye. It consists primarily of three layers: an outer layer of epithelium, a middle stromal layer of collagen-rich extracellular matrix (ECM) interspersed with keratocytes and an inner layer of endothelial cells. The stroma comprises 90% of the thickness of the cornea. It consists of dense, regularly packed collagen fibrils arrange as orthogonal layers or lamellae. The corneal stroma is unique in having a homogeneous distribution of fibrils with small diameter (25–30 nm) that are regularly packed within lamellae and this arrangement minimizes light scattering permitting transparency. When an incisional wound penetrating into stroma occurs, the keratocytes become hypercellular myofibroblasts. These cells can later become wound fibroblast, which provides continued transparency or become myofibroblasts that produce a disorganized ECM resulting in corneal opacity [1].

The limbus of the eye, located at the junction of the cornea and conjunctiva of the ocular surface, represents a unique stem cell niche in human body. The adult corneal epithelium is continuously regenerated from stem cells (SC) located at the basal layer of the limbal epithelium. The limbal epithelial stem cells differ from corneal epithelium in their lack of expression of corneo-specific differentiation keratins (K3/K12) [2-4], connexin 43-mediated gap junction intercellular communication [5-7], and the nuclear transcription factor p63 [8,9], cell cycle length [10], and label retaining property [11]. One important mechanism that modulates the ‘stemness’ characteristic of the limbal SC is that the limbal stroma provides a unique microenvironment or niche that is strategically protected by heavy pigmentation and is highly innervated and vascularized. Clinically, destruction of limbal epithelial SC or the limbal stromal niche can lead to a pathological stage of limbal SC deficiency with severe loss of vision [12]. Chronic inflammation in the limbal deficient stroma is sufficient to cause detrimental damage to the conjunctival limbal autograft transplanted to patients [13] or experimental rabbits [14]. These findings suggested that the limbal stromal niche is critical in regulating the self-renewal and the fate of SC, although the mechanism remains elusive. Study had shown that the limbal stroma modulates epithelial differentiation, proliferation and apoptosis in the direction favoring stemness [15]. Intriguingly however, one report has shown that the limbal microenvironment was able to induce transdifferentiation of hair follicle stem cells into corneal epithelial-like cells [16].

Embryonic stem cells (ESC) are cells derived from the epiblast tissue of the inner cell mass (ICM) of a blastocyst or earlier morula stage embryos. A blastocyst consisting of 50–150 cells is an early stage embryo-approximately four to five days old in humans. ESC are pluripotent and give rise during development to all derivatives of the three primary germ layers: ectoderm, endoderm and mesoderm. In other words, they can develop into each of the more than 200 cell types of the adult body when given sufficient and necessary stimulation for a specific cell type. They do not contribute to the extra-embryonic membranes or the placenta. A human embryonic stem cell is also defined by the presence of several transcription factors and cell surface proteins. The transcription factors octamer-binding transcription factor 4 (Oct4), Nanog, and SRY (sex determining region Y)-box 2 (Sox2) form the core regulatory network that ensures the suppression of the genes that lead to differentiation and the maintenance of pluripotency [17]. The cell surface antigens most commonly used to identify ESC are the glycolipids stage-specific embryonic antigen 3 (SSEA-3) and stage-specific embryonic antigen 4 (SSEA-4) and the keratan sulfate antigens Tra-1–60 and Tra-1–81.

Recently, the existence of multipotent differentiation cells in limbal stroma was reported. An ATP-binding cassette sub-family G member 2 (ABCG2)-expressing cell population from limbal stroma which was isolated as a side population by cell sorting was able to differentiate into chondrocytes and neurons following induction [18]. In other studies, MSC-like multipotent cells were also found in corneal stroma [19] and limbal stroma [20]. More interestingly, an isolated population of limbal stromal cells which expressed SSEA-4 was reported to express a panel of ESC markers and demonstrated multilineage differentiation potential [21]. Yet, despite the potential use of the multipotent cells in cell-based therapy and corneal tissue engineering, further studies are needed to support the findings as some of the studies have not been reproduced and to our knowledge there is only one report for reference [21]. Thus, we study the expression of SSEA-4 and other embryonic stem cell markers such as Oct-4, Nanog, Sox2, Tra-1–60, and Tra-1–81 besides focus on the limbal stromal cells characteristic and their multipotential that mimic MSC. This is important as expression of SSEA-4 and other embryonic stem cells markers will reveal the transdifferentiation potential of these cells toward ESC and their future application in regenerative medicine.

## Methods

The research protocol was approved by the Medical Research and Ethics Committee, Ministry of Health and the Medical Research Secretariat, Universiti Kebangsaan Malaysia.

### Establishment of limbal stromal cell culture

Corneoscleral rims from cadaveric donors were obtained post cornea graft transplantation with informed consent from donor’s relative. The rims were washed with phosphate buffer saline (PBS; Invitrogen Corporation, Carlsbad, CA) and then trimmed to remove the sclera. The limbal tissues were incubated at 37 °C for 2h with dispase (BD Biosciences, Mississauga, Canada) at a concentration of 5 mg/ml. After washing with PBS, the limbal tissues were then cut into approximately 2 mm in size and cultured on matrigel (BD Biosciences) coated plate with complete medium modified from Dravida et al. [21] containing Dulbecco’s Modified Eagle’s Medium (DMEM)/F12, 10% knockout serum, 10 µg/ml insulin, 5 µg/ml transferrin, 5 µg/ml selenium-X, 100 IU/ml penicillin, 100 µg/ml streptomycin (all from Invitrogen Corporation, Carlsbad, CA), 10 ng/ml leukemia inhibitory factor (LIF; Sigma-Aldrich Chemic, Steinheim, Germany) and 4 ng/ml basic fibroblast growth factor (bFGF; BD Biosciences). Spindle cell-like outgrowths were cultured for three to four weeks until near confluent. The spindle cells were called limbal stromal cells. These cells were then trypsinized and plated on matrigel coated flasks. The cultures were maintained in 5% CO_2_ in a humidified incubator at 37 °C. When the cells reached 80%–90% confluence, the cells were harvested with 0.25% trypsin-EDTA (Invitrogen Corporation) and subcultured.

### Corneal epithelial cell culture

Corneal epithelial cell line (American Type Culture Collection, [ATCC], Manassas, VA) was propagated and cultured according to manufacturer’s protocol. Total RNA of nearly confluent corneal epithelial cells were extracted for reverse transcription polymerase chain reaction (RT–PCR).

### Human embryonic stem cell (hESC) culture

Embryonic stem cell line, BG01V (ATCC) at passage 16 were cultured on mitomycin treated MEF cells (ATCC) with 80% knockout DMEM supplemented with 20% Gibco knockout SR, 1% MEM-non essential amino acid (NEAA), 1% Glutamax, 0.1 mM B-mercaptoethanol (BME), 10 IU/ml penicillin, 10 µg/ml streptomycin (all from Invitrogen Corporation), and 4 ng/ml human basic fibroblast growth factor (bFGF; BD Biosciences) [22]. Cell colonies at 70% confluence were harvested with collagenase and gently scrapped with 5 ml serological pipette. The hESC pellets were washed once with culture medium and resuspended in mTeSR^TM^ medium (Stem Cell Technologies Inc., Vancouver, Canada). The cell pellets were cultured on six-well plates coated with matrigel (BD Biosciences) as per manufacturer’s protocol. The hESC were subjected to flow cytometric analysis and total RNA extraction when ready to passage.

### Corneal stromal cell culture

Cryopreserved corneal stromal cells, courtesy of Choong et al. [19] were cultured with DMEM low glucose medium supplemented with 20% fetal bovine serum (FBS), 100 IU/ml penicillin and 100 µg/ml streptomycin (all from Invitrogen Corporation). The cells at 80% confluence were subjected to flow cytometric analysis and total RNA extraction when ready to passage.

### Cell sorting

Trypsinized passage-2 (P2) cells were incubated at 1.0×10^6^ cells per ml in PBS with 2% FBS and 20 µl SSEA-4- fluorescein isothiocynate (FITC) conjugated monoclonal antibody (BD Biosciences) for 30 min at 4 °C. After staining, the cells were washed twice in PBS with 2% FBS and then stored in PBS with 2% FBS on ice. Cells were analyzed on a FASC Aria II (BD Biosciences) high-speed cell sorter using the 488 nm excitation and 100 µm nozzle. Sorted positive, SSEA-4^+^ cells and negative, SSEA-4^-^ cells were collected and cloned at 1×10^4^ cells per cm^2^ as mentioned above. When nearly confluent, the cells were harvested for the re-analysis of SSEA-4 expression and subsequent experiments.

### Flow cytometry analysis

The limbal stromal cells were stained with multiple fluorescein conjugated antibodies against a panel of mesenchymal markers (cluster of differentiation [CD]90, CD71, CD73, CD29, CD44, CD105, CD123, CD271 and human major histocompatibility class II receptor encoded by human leukocyte antigen [HLA-DR]), hematopoietic markers (CD34, CD117, CD45 and CD14), human embryonic stem cell markers (SSEA-1, SSEA-4, Tra-1–60, Tra-1–81, Oct 3/4, Nanog and Sox2). All antibodies were from BD Biosciences, and putative stem cell marker ABCG2. The expression of ABCG2 and mesenchymal markers by limbal stromal cells was also compared to the corneal stromal cells. Briefly, a single cell suspension of limbal stromal cells (0.5–1×10^6^ cells each) at passage 2, in 100 μl of PBS supplemented with 2% fetal bovine serum (FBS; Invitrogen Corporation), was incubated with 20 μl of fluorescein conjugated antibodies for 30 min at 4 °C. After two washes, the cells were suspended in 1 ml of PBS supplemented with 2% FBS. Stained cells were subjected to flow cytometric acquisition with FACS Caliber instrument (Becton Dickinson [BD], Heidelberg, Germany) and a total of 10,000 events were acquired for data analysis by using Cell Quest software (BD, San Jose, CA). An isotype control was included in each experiment to exclude data from non-specific binding.

### Immunocytochemistry

The expression of markers such as SSEA-4, ABCG2, p63 (Millipore, Billerica, MA), Pax 6, corneal epithelium related cytokeratin 3 (AE5; Santa Cruz Biotechnology, Inc., Santa Cruz, CA), cytokeratin 10 (CK10; DakoCytomation Inc., Carpinteria, CA), vimentin, a-smooth muscle actin (α-SMA; Millipore, Billerica, MA) and corneal stromal proteoglycan protein-keratocan (Santa Cruz Biotechnology, Inc.) were studied by immunocytochemical staining. Limbal stromal cells at P2–6 were cultured on chamber slides prior fixation with fresh 4% paraformaldehye at room temperature for 20 min. After washing with PBS for three times, the cells were permeabilized with 0.5% Triton X-100 for 5 min. The cells were washed three times with PBS before blocking with 1% bovine serum albumin (BSA) for 30 min and then incubated with primary antibodies diluted in 1% BSA (1:100) for 1 h followed by another washing with PBS. Fluorescein isothiocynate (FITC) conjugated secondary antibody (1:100; Millipore) was applied for 1 h and the tissue was counterstained with DNA binding dye propidium iodide for 5 min or 4',6-diamidino-2-phenylindole (DAPI). The slide chambers were mounted with fluorescence mounting medium (DakoCytomation Inc., Carpinteria, CA) using a coverslip. The slides were examined under a fluorescence microscope (Nikon Corporation, Chiyoda-ku, Tokyo, Japan).

### Adipogenic and osteogenic differentiation

Passage-3 limbal stromal cells were seeded at 5×10^4^ per cm^2^ in 35 mm culture dish and cultured with adipogenic and osteogenic medium prepared according to established methods [23]. These cells were induced for 21 days with the medium being changed on every alternate day. After 21 days, the adipogenic and osteogenic cultures were fixed and stained with Oil Red O (0.3%) and Alizarin Red solution. The stained cells were examined under an inverted microscope immediately after staining. Expression of lipoprotein lipase (LPL) and osteocalcin was assessed by RT–PCR.

### Chondrogenic differentiation

Limbal stromal cells at P3 were diluted to a final concentration of 2.5×10^5^ cells/ml in DMEM media supplemented with 10% FBS. One ml of the suspension was transferred to a 15 ml polypropylene conical tube and centrifuge for 5 min at 150× g at room temperature. The supernatant was discarded completely and 1 ml of pre-warmed chondrocyte differentiation medium (Miltenyi Biotec GmbH, Bergisch, Gladbach, Germany) was added to resuspend the cells. The centrifugation step was repeated to obtain cell pellet. The cell pellet in the centrifuge tube was incubated with the chondrocyte medium in CO_2_ incubator with 5% CO_2_ and >95% humidity. On day 24, the cells nodule was rinsed and embedded in optimal cutting temperature (OCT) compound for 10 µm sectioning with a cryostat (Leica Biosystems Nussloch GmbH, Nussloch, Germany). The cryosections were fixed in 4% buffered paraformaldehyde for 5 min and stained with alcian blue (pH 1.0) for the detection of cartilage matrix [23]. Reverse transcription polymerase chain reaction (RT–PCR) was used to analyze cartilage-specific genes such as collagen II and aggrecan (Table 1).

**Table 1:** Human primer sequences used for RT–PCR.

**Gene**	**Accession**	**Sense primer**	**Antisense primer**	**Product size (bp)**
SSEA-4	NM_006927	TGGACGGGCACAACTTCATC	GGGCAGGTTCTTGGCACTCT	118
ABCG2	AY017168	AGTTCCATGGCACTGGCCATA	TCAGGTAGGCAATTGTGAGG	379
ΔNp63	XM_036421	CAGACTCAATTTAGTGAG	AGCTCATGGTTGGGGCAC	440
Pax 6	NM_00028	ATAACCTGCCTATGCAACCC	GGAACTTGAACTGGAACTGAC	208
AE-5	NM_057808	CTACCTGGATAAGGTGCGAGCT	TCTCGCATTGTCAATCTGCA	150
Keratocan	NM_007035	ATCTGCAGCACCTTCACCTT	CATTGGAATTGGTGGTTTGA	167
LPL	XM_044682	GAGATTTCTCTGTATGGCACC	CTGCAAATGAGACACTTTCTC	276
Osteocalcin	X53698	ATGAGAGCCCTCACACTCCTC	GCCGTAGAAGCGCCGATAGGC	294
Collagen II	NM_001844	TTTCCCAGGTCAAGATGGTC	CTTCAGCACCTGTCTCACCA	377
Aggrecan	X17406	TGAGGAGGGCTGGAACAAGTACC	GGAGGTGGTAATTGCAGGGAACA	350
GAPDH	M33197	GCCAAGGTCATCCATGACAAC	GTCCACCACCCTGTTGCTGTA	498

The table details the primers and sequences, their accession and expected product size after RT-PCR.

### Reverse transcription polymerase chain reaction (RT–PCR) analysis

Expression of embryonic protein SSEA-4 by limbal stromal cells was compared to hESC, BG01V. Other transcripts related to limbal epithelial phenotype such as ABCG2, p63, Pax6; corneal epithelium related cytokeratin AE-5, corneal stromal proteoglycan protein-keratocan sulfate were also evaluated. Cultured corneal stromal and epithelial cells were also tested for these markers simultaneously. To assess the differentiation potential, expression of genes related to adipocytes (LPL), osteocytes (osteocalcin) and chondrocytes (collagen II and aggrecan) was also evaluated. Total RNA was extracted from limbal stromal cells and keratocytes near confluency at P3 using RNAeasy kit (Qiagen Hamburg GmbH, Hamburg, Germany) according to the manufacturer’s protocol. The extracted RNA was quantified by reading the absorbance at 260 nm, and its purity was evaluated from the 260/280 ratio of absorbance with spectrophotometer (BioPhotometer Plus, Eppendorf, Hamburg, Germany). First strand cDNA was synthesized with Transcriptor First Strand cDNA synthesis kit (Roche Applied Science, Nonnenwald, Penzberg, Germany) as per protocol. Touchdown PCR were performed with primers (Table 1) and PCR kit (Qiagen Hamburg GmbH, Hamburg, Germany) on a thermocycler (Eppendoff Mastercycler gradient, Hamburg, Germany). Initial denaturation was started at 95 °C for 5 min, followed by denaturation at 94 °C for 30 s, annealing at 65 °C for 15 s, and extension at 72 °C for 30 s. The reactions were repeated with decrement of annealing temperature at 1 °C every cycle for 15 cycles. For subsequent reactions, denaturations were fixed at 94 °C for 30 s, and then annealing at 50 °C for 15 s, and extensions at 72 °C for 30 s for a total of 23 cycles. A final extension of 5 min at 72 °C was also performed for each reaction. The PCR products were analyzed on 1.5% agarose gel and scanned using an ultraviolet (UV) gel doc (Bio-Rad, Hercules, CA). The expression of various markers was normalized using GAPDH as an internal control.

## Results

**Figure 1:**
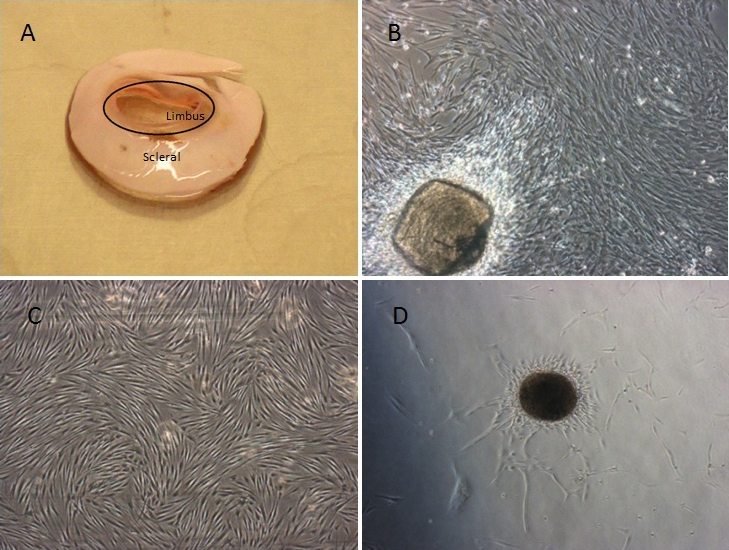
Morphological observations. One of the corneoscleral rim used in the study. The black circle shows where the corneoscleral rim was trimmed to separate the limbal explant from the whitish scleral tissue (**A**). Phase contrast microscopic shows the outgrowth of limbal stromal cells from the limbal explants on day 7 (magnification: 40×; **B**). Confluent culture of limbal stromal cells shows spindle morphology with petal growing pattern (magnification: 40×; **C**); Sphere formation by the limbal stromal cells when cultured with complete media without matrigel (**D**).

The limbal stromal cells were established from corneoscleral rims tissues (Figure 1A) and cultured as described previously [21]. Cells outgrowth were observed after a few days of plating (Figure 1B) and the cells reached confluence in about 3–4 weeks. The limbal stromal cells appeared to be fibroblastic, elongated, spindle shape with a petal-like growing pattern (Figure 1C). These cells were able to form colonies with occasional cell spheres formation which resemble embryoid bodies (Figure 1D). The stromal cells could be cultured up to 13 passages or more. Three derived limbal stromal cells were used in the subsequent experiments.

### Cell sorting

**Figure 2:**
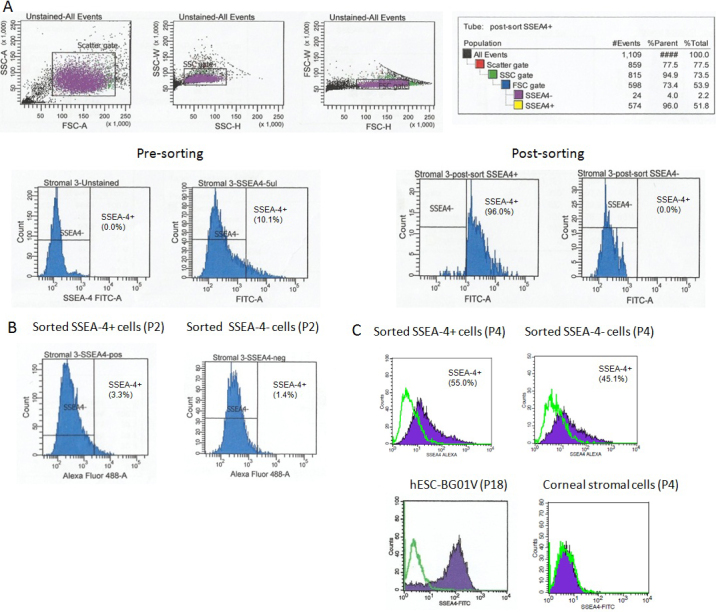
Flow cytometric analysis of SSEA-4 expression in cultured limbal stromal cells. **A**: Isolation of SSEA-4^+^ and SSEA-4^-^ limbal stromal cells at passage (P) 1 by FACS. Cell debris and doublets were discriminated before cell sorting as shown in the first three panels. Gating hierarchy was shown in the table. Percentages of pre- and post-sorted cells are also depicted in histograms. The sorted SSEA-4^+^ and SSEA-4^-^ cells were cultured separately. **B**: Propagated cells after cell sorting were re-analyzed for the expression of SSEA-4 at P2 and P4 (**C**). Expression of SSEA-4 in hESC is also demonstrated. The green line in the histograms represents the isotype control.

The expression of embryonic marker SSEA-4 by the limbal stromal cells were studied and we found a small population (0.1% - 10.1%) of limbal stromal cells showed positive expression of SSEA-4 (SSEA-4^+^). Dot plots of representative derived limbal stromal cells were shown (Figure 2A). The SSEA-4^+^ cells and SSEA-4^-^ cells were sorted into matrigel coated six-well plate and cultured with complete culture medium. Clonal expanded cells from the sorted cells were harvested and subjected to FACS analysis for SSEA-4 expression. The results showed that the clonal expanded SSEA-4^+^ cells and SSEA-4^-^ had almost similar and low expression of SSEA-4 i.e., 3.3% and 1.4% (Figure 2B). Our results did not show increase of SSEA-4^+^ population after purification and expansion followed by cell sorting. However, the SSEA-4^+^ cells from the sorted SSEA-4^+^ and SSEA-4^-^ were increased in subsequent cultures to 55.0% and 45.1% respectively at P4 (Figure 2C). When compared to hESC, the expression intensity of SSEA-4 in limbal stromal cells was much lower. We also compared the expression of SSEA-4 with corneal stromal cells and our results showed that the cells did not express the protein.

### Flow cytometry

**Figure 3:**
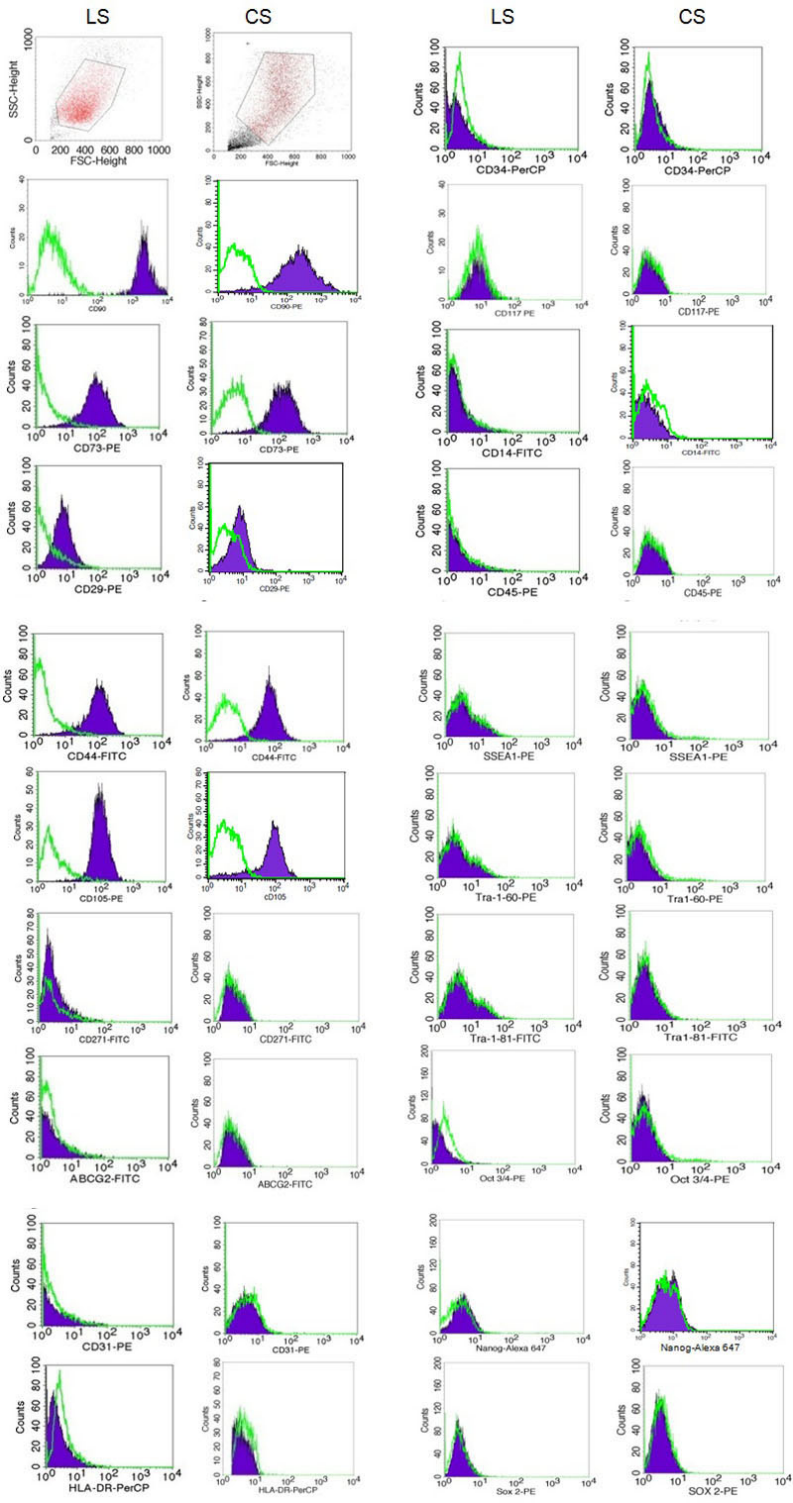
Expression profiles of limbal stromal (LS) at passage 6 and corneal stromal (CS) cells at passage 4 by FACS analysis. The two populations of cells have very similar expression where they expressed mesenchymal markers and absence of hematopoietic markers and endothelial marker (CD31). The cells did not express ABCG2 and other embryonic stem cells markers. The green line in the histograms represents the isotype controls.

Derived limbal stromal cells from P2–6 were subjected to FACS analysis and compared to the expression profile of corneal stromal cells (Figure 3). The cells expressed mesenchymal markers such as CD90, CD73, CD29, CD44, CD105 but lack expression of CD71, CD271 (NGFR), HLA-DR, and endothelial marker, CD31. Besides, the cells showed negative expression of hematopoietic markers such as CD34, CD117 (c-kit), CD45, and CD14. Negative expression of ABCG2 and embryonic markers such as SSEA1, Tra-1–60, Tra-1–81, Oct 3/4, Nanog, and Sox2 was also observed. Table 2 summarizes the immunophenotyping results by FACS analysis.

**Table 2:** Immunophenotyping of limbal stromal and corneal stromal cells by FACS analysis.

**No.**	**Surface marker**	**Limbal stromal cells ± (%)**	**Corneal stromal cells ± (%)**
1	CD90	+ (100±0)	+ (61±4)
2	CD73	+ (99±1)	+ (97±2)
3	CD29	+(29±6)	+ (76±7)
4	CD44	+ (90±5)	+ (96±3)
5	CD105	+ (92±6)	+ (94±4)
6	CD71	− (0)	+ (7±3)
7	CD271	− (0)	− (0)
8	HLA-DR	− (0)	− (0)
9.	CD31	− (0)	− (0)
10	CD34	− (0)	− (0)
11	CD117	− (0)	− (0)
12	CD14	− (0)	− (0)
13	CD45	− (0)	− (0)
14	ABCG2	− (0)	− (0)
15	SSEA-1	− (0)	− (0)
16	Tra-1–60	− (0)	− (0)
17	Tra-1–81	− (0)	− (0)
18	Oct 3/4	− (0)	− (0)
19	Nanog	− (0)	− (0)
20	Sox 2	− (0)	− (0)

The table summarizes the results of immunophenotyping of limbal stromal and corneal stromal cells by FACS analysis for MSC, hematopoietic and ESC markers. Percentage of expression of a marker is given in brackets (average values of three such experiments ±standard deviation). The symbol (−) indicates negative expression while the symbol (+) indicates positive expression of a marker.

### Immunocytochemistry

**Figure 4:**
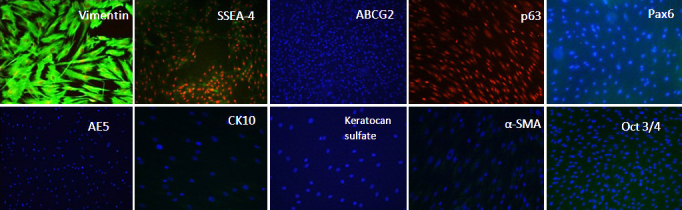
Immunofluorescence staining of limbal stromal cells. The limbal stromal cells were positive for vimentin (200×; green), but dim positive for SSEA-4 (100×; green). Expression for ABCG2 (100×), p63 (100×), Pax 6 (100×), AE5 (100×), CK 10 (200×), keratocan sulfate (200×), α-SMA (200×), and Oct 3/4 (100×) proteins was found negative. The nuclei were counterstained either with propidium iodide (red) or DAPI (blue).

Results for immunofluorescence staining were shown in Figure 4. The limbal stromal cells were stained negative for p63, cytokeratin 3 (AE5), and CK10 which ruled out the possibility of corneal epithelial and conjunctival cells contamination in the cultures. Bright positive expression of vimentin confirmed the mesenchymal phenotype of the cells. Pax6 is a homeobox transcription factor expressed in embryonic ocular precursor cells and epithelial cells but absent in adult keratocytes [24]. We found that Pax6 was absent in the stromal cells as well as ABCG2 transporter protein when analyzed by immunofluorescence staining. The absence of corneal stromal proteoglycan protein-keratocan sulfate revealed the activated stromal phenotype of the cells. However the cells were stained negative with α-SMA, which excluded the myofibroblast phenotype of the limbal stromal cells. The stromal cells were dim positive for SSEA-4 and negative for Oct 3/4 as similar to the results shown by FACS analysis.

### Diffferentiation study

**Figure 5:**
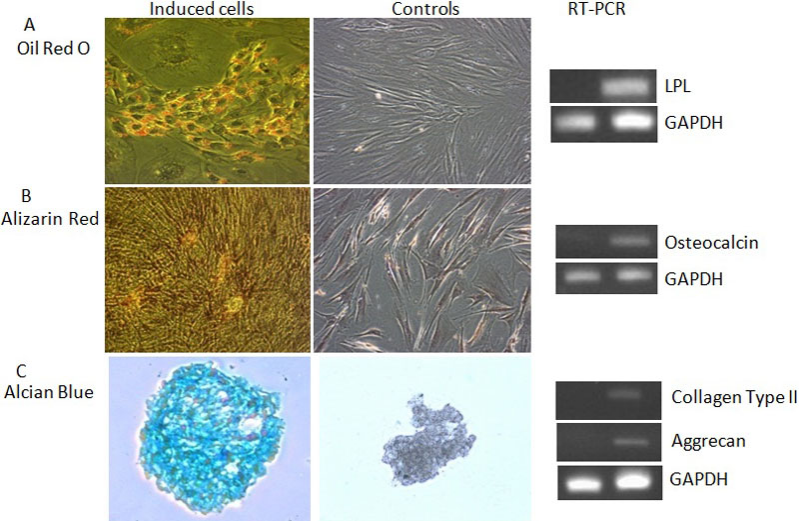
Differentiation of human limbal stromal cells. **A**: Adipogenic differentiation could be induced as examined by Oil Red-O staining (magnification 200×). **B**: Osteogenic differentiation (100×). **C**: Chondrogenic differentiation (50×) as examined by Alizarin Red and Alcian Blue staining; The results of RT–PCR for the relevant transcripts after differentiation were also depicted (left lanes: uninduced cells; right lanes: induced cells).

The derived limbal stromal cells differentiated into adipocytes, osteocytes and chondrocytes when induced (Figure 5). About 46% of the limbal stromal cells were able to differentiate into adipoctyes as shown by the red droplets stained by Oil Red O (Figure 5A). However, we noticed that only early passage of the cells i.e P2–3 had the capability to differentiate into adipocytes. The expression of adipogenesis specific transcripts such as lipoprotein lipase (LPL) was positive as compared to un-induced cells.

For osteogenesis, the confluent layer of induced cells appeared orange-red when stained with Alizarin Red (Figure 5B). The un-induced cells were stained negative with Alizarin Red. The induced cells expressed osteogenesis-specific transcripts such as osteocalcin as compared to un-induced cells.

For chondrogenesis, alcian blue at pH 1.0 was used to stain the cryosections of induced cell pellets. The sections were stained blue as compared to un-induced cell pellets (Figure 5C). RT–PCR result showed that the chondrogenic pellet expressed collagen type II and aggrecan whereas un-induced cell pellets did not express the respective mRNA.

### RT–PCR analysis

**Figure 6:**
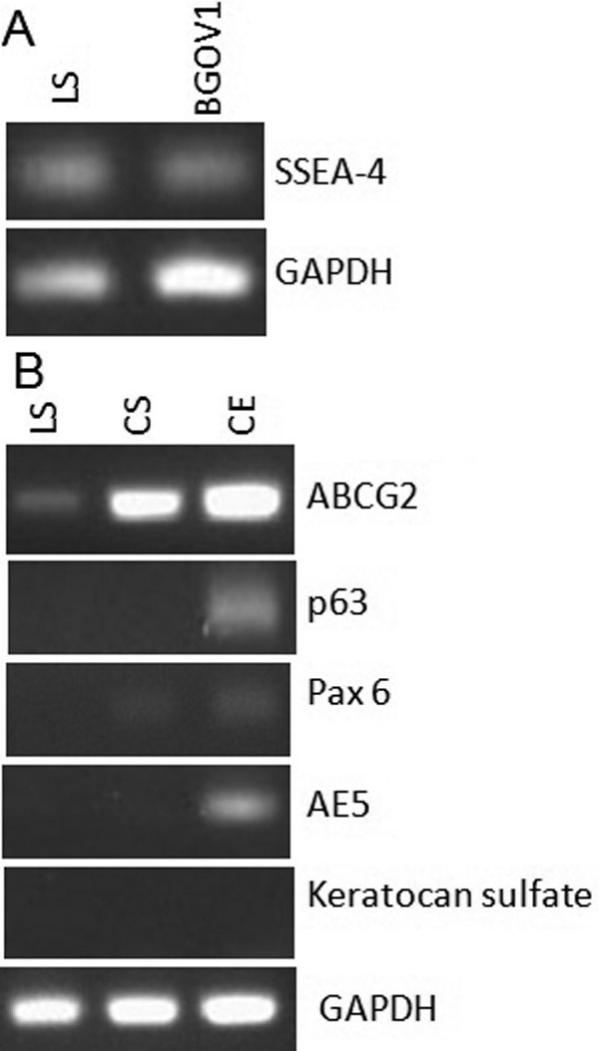
Analysis of reverse transcriptase polymerase chain reactions (RT–PCR) of limbal stromal cells. **A**: Expression of SSEA-4 transcripts by limbal stromal (LS) cells as compared to human embryonic stem cells, BG01V. **B**: Expression of various transcripts by limbal stromal cells such as ABCG2, p63, Pax 6, AE5, and keratocan sulfate was compared to corneal stromal (CS) and corneal epithelial (CE) cells. GAPDH was served as housekeeping gene.

Our results confirmed the expression of SSEA-4 in the limbal stromal cells as compared to hESC, BG01V (Figure 6A). One of the transcripts related to limbal epithelial phenotype i.e., ABCG2 was expressed by all the three cell types: limbal stromal cells, corneal stromal and corneal epithelial cells (Figure 6B). However, the expression of this transporter protein was not detected by flow cytometry analysis and immunocytochemistry study (Figure 3 and Figure 4). Other transcripts such as p63, Pax6 and cytokeratin 3 (AE5) were present in corneal epithelial cells but absent in limbal stromal cells whereas corneal stromal proteoglycan protein-keratocan sulfate was absent in both limbal stromal and corneal stromal cells. These results were consistent with those from immunocytochemistry study.

## Discussion

In this study, we derived limbal stromal cells and investigated the expression of SSEA-4 surface protein and other ESC markers on these cells. We also studied the MSC characteristic, phenotypes and multipotent differentiation potential of these cells toward adipocytes, osteocytes, and chondrocytes.

Our first finding confirmed that a sub-population of ex vivo expanded limbal stromal cells expressed SSEA-4 surface protein as shown by flow cytometric analysis, immunostaining and RT–PCR. However, the limbal stromal cells did not express other markers for hESC such as Tra1–60, Tra-1–81, Oct 3/4, Nanog, and Sox2 as shown by flow cytometric analysis. We also found that the derived limbal stromal cells exhibited the characteristic of mesenchymal stromal cells as supported by flow cytometric analysis with mesenchymal markers, immunofluorescence staining with vimentin and by observation of its differentiation potential.

The expression of SSEA-4 in limbal stromal cells or limbal fibroblast had only been reported by Dravida et al. [21]. The authors were able to isolate the SSEA-4^+^ cells by magnetic beads purification technique. Although our post-sorting analysis showed that only positive cells were sorted, but the purifications of the SSEA-4^+^ cells were unsuccessful as shown by the percentage of propagated SSEA-4^+^ cells at passage 2. This outcome might be due to the loss of SSEA-4 expression in the sorted cells following culture. This was possible as the expression of SSEA-4 in these cells was close to the negative control peak and very low intensity was observed as compared to the higher expression of SSEA-4 in hESC. The low expression of SSEA-4 was also found in bone marrow, adipose tissue, heart and dermis stromal cells [25,26]. However at passage 4 and subsequent cultures, the percentage of SSEA-4^+^ cells was markedly increased. Some propagated cells became SSEA-4^+^ after multiple subcultures. Thus, further study is needed to find out the cause of this outcome. The SSEA-4 antibodies (clone MC813–70) however were reported to be non-specific as it also binds to nonsphingoid molecules such as glycoproteins which carry an epitope recognized by SSEA-4 antibodies [27]. This could be the reason as the increase of SSEA-4 transcript was not detected by RT–PCR despite the increase percentage of SSEA-4^+^ cells. Besides, the expression of other hESC markers such as Tra-1–60, Tra-1–81, Oct 3/4, Nanog and Sox2 were not detected in multiple subcultured cells. This finding was consistent with the findings from Brimble et al. that the SSEA-4 is not essential for hESC pluripotency. Therefore, we infer that the SSEA-4 detected in these cells might be different from that detected in hESC.

A previous study showed that the limbal stromal cells expressed markers of mesenchymal stromal cells [20]. Our results concurred with the finding and also demonstrated that the corneal stromal cells had similar expression profile compared to the limbal stromal cells except for the expression of SSEA-4. While this observation might be due to the differences of culture medium that we used for limbal and corneal stromal cells, the comparison gives us the clue whether the markers are expressed in the expanded corneal stromal cells. One of our important observations was that not all bone marrow MSC markers such as CD71 and CD217 were expressed in these cells. The results of our study had also provided added evidence on the differentiation capability of these cells toward adipogenesis, osteogenesis and chondrogenesis, but only at early passage 2 and 3. In addition, we also noticed the differentiation potential of these cells was poorer than the bone marrow mesenchymal stromal cells (unpublished). Thus, despite the increased expression of SSEA-4 which was detected by flow cytometry, the differentiation potential of the cells was not increase. This result was different from that reported by Gang et al. where SSEA-4^+^ cells have higher potential of proliferation and differentiation. This might be caused by different proliferation and differentiation potentials among the bone marrow stromal and limbal stromal cells.

Our results showed that the derived limbal stromal cells in this study were different from that of the purified side population cells as reported by Du et al. The side population of cells from corneal stroma had not only expressed ABCG2 and Pax6 but they were able to differentiate into inactivated keratocytes which produced keratocan sulfate protein. In our study, except for vimentin, these proteins and α-SMA were not detected by immunostaining. Therefore, the derived limbal stromal cells exhibited mesenchymal/fibroblast-phenotype but not that of myofibroblast. The difference between our findings and theirs might be due to the different isolation and culture methods used. The side population cells might represent a more primitive undifferentiated stem or progenitors as compared to the explant culture method that we applied. Nevertheless, the method we had applied was simply straight forward and easy to follow. The culture method that we adapted from Dravida et al. [21] was also used to culture embryonic stem cells without the support of feeder layer cells. Matrigel, an extra cellular matrix was used as a substitute for the feeder cells. However, as shown by our results, this culture system was not able to induce the limbal stromal cells to transdifferentiate into embryonic-like cells but produced cells with mesenchymal stromal phenotype. With the emerging knowledge of induced pluripotent stem cells (iPSC), there are more effective ways to transdifferentiate stromal cells to pluripotent cells [28,29].

Our findings provide new insight to the expression of SSEA-4 in limbal stromal cells. Although another study [21] reported that the SSEA-4^+^ limbal stromal cells also expressed other embryonic specific markers and transcription factors, we did not find the same population of cells in our study even though we adapted the same culture system. Thus, our study highlights the limited plasticity of limbal stromal cells such as their trandifferentiation potential which mimic embryonic stem cells. This study has proven that SSEA-4 might not be a good marker for the enrichment of cells with embryonic-like property. This is important as many investigators have thought that cells expressing SSEA-4 have the characteristic of embryonic stem cells and they might want to pursue a similar study.

In summary, we report here that an expanded limbal stromal cell with a fibroblastic phenotype, expressed SSEA-4 but not other embryonic stem cell markers. These cells expressed majority but not all MSC markers and demonstrated multipotentiality toward adipocytes, osteocytes and chondrocytes. Since different types of expanded limbal stromal cells are reported, depending on the isolation and culture methods, we postulate that the limbus may consist of stromal cells with different maturity. Thus, future studies that focus on the phenotype and characteristic of limbal stromal cells in situ would be necessary to confirm and identify the different population of limbal stromal cells. The potential of these cells in regenerative medicine especially for cornea repair shoud be tested in an animal model. Besides, it would be interesting to study iPSC that able to differentiate into corneal/limbal epithelial or stromal cells.
